# Comparative Efficacy of Transsphenoidal and Transcranial Approaches for Treating Tuberculum Sellae Meningiomas: A Systematic Review and Meta-Analysis

**DOI:** 10.3390/jcm13082356

**Published:** 2024-04-18

**Authors:** Edoardo Agosti, A. Yohan Alexander, Sara Antonietti, Marco Zeppieri, Amedeo Piazza, Pier Paolo Panciani, Marco Maria Fontanella, Carlos Pinheiro-Neto, Tamara Ius, Maria Peris-Celda

**Affiliations:** 1 Division of Neurosurgery, Department of Medical and Surgical Specialties, Radiological Sciences and Public Health, University of Brescia, Piazza Spedali Civili 1, 25123 Brescia, Italy; edoardo_agosti@libero.it (E.A.);; 2 Department of Neurologic Surgery, Mayo Clinic, Rochester, MN 55905, USA; 3 Department of Ophthalmology, University Hospital of Udine, p.le S. Maria della Misericordia 15, 33100 Udine, Italy; 4 Department of Neurosurgery, “Sapienza” University, 00185 Rome, Italy; 5 Department of Otorhinolaryngology, Mayo Clinic, Rochester, MN 55905, USA; 6 Neurosurgery Unit, Head-Neck and NeuroScience Department, University Hospital of Udine, p.le S. Maria della Misericordia 15, 33100 Udine, Italy

**Keywords:** tuberculum sellae meningioma, microsurgical transcranial, endoscopic endonasal, systematic reviews, meta-analysis, outcomes

## Abstract

**Background/Objectives**: Tuberculum sellae meningiomas (TSMs) constitute 5–10% of intracranial meningiomas, often causing visual impairment. Traditional microsurgical transcranial approaches (MTAs) have been effective, but the emergence of innovative surgical trajectories, such as endoscopic endonasal approaches (EEAs), has sparked debate. While EEAs offer advantages like reduced brain retraction, they are linked to higher cerebrospinal fluid leak (CSF leak) risk. This meta-analysis aims to comprehensively compare the efficacy and safety of EEAs and MTAs for the resection of TSMs, offering insights into their respective outcomes and complications. **Methods**: A comprehensive literature review of the databases PubMed, Ovid MEDLINE, and Ovid EMBASE was conducted for articles published on TSMs treated with either EEA or MTA until 2024. The systematic review was performed according to the Preferred Reporting Items for Systematic Reviews and Meta-Analysis guidelines. Meta-analysis was performed to estimate pooled event rates and assess heterogeneity. Fixed- and random-effects were used to assess 95% confidential intervals (CIs) of presenting symptoms, outcomes, and complications. **Results***:* A total of 291 papers were initially identified, of which 18 studies spanning from 2000 to 2024 met the inclusion criteria. The exclusion of 180 articles was due to reasons such as irrelevance, non-reporting of selected results, systematic literature review or meta-analysis, and a lack of details on method/results. The 18 studies comprised a total sample of 1093 patients: 444 patients who underwent EEAs and 649 patients who underwent MTAs for TSMs. Gross total resection (GTR) rates ranged from 80.9% for EEAs to 79.8% for MTAs. The rate of visual improvement was 86.6% in the EEA group and 65.4% in the MTA group. The recurrence rate in the EEA group was 6.9%, while it was 5.1% in MTA group. The postoperative complications analyzed were CSF leak, infections, dysosmia, intracranial hemorrhage (ICH), and endocrine disorders. The rate of CSF leak was 9.8% in the EEA group and 2.1% in MTA group. The rate of infections in the EEA group was 5.7%, while it was 3.7% in the MTA group. The rate of dysosmia ranged from 10.3% for MTAs to 12.9% for EEAs. The rate of ICH in the EEA group was 0.9%, while that in the MTA group was 3.8%. The rate of endocrine disorders in the EEA group was 10.8%, while that in the MTA group was 10.2%. No significant difference was detected in the rate of GTR between the EEA and MTA groups (OR 1.15, 95% CI 0.7–0.95; *p* = 0.53), while a significant benefit in visual outcomes was shown in EEAs (OR 3.54, 95% CI 2.2–5.72; *p* < 0.01). There was no significant variation in the recurrence rate between EEA and MTA groups (OR 0.92, 95% CI 0.19–4.46; *p* = 0.89). While a considerably increased chance of CSF leak from EEAs was shown (OR 4.47, 95% CI 2.52–7.92; *p* < 0.01), no significant difference between EEA and MTA groups was detected in the rate of infections (OR 1.92, 95% CI 0.73–5.06; *p* = 0.15), the rate of dysosmia (OR 1.25, 95% CI 0.31–4.99; *p* = 0.71), the rate of ICH (OR 0.61, 95% CI 0.20–1.87; *p* = 0.33), and the rate of endocrine disorders (OR 1.16, 95% CI 0.69–1.95; *p* = 0.53). **Conclusions**: This meta-analysis suggests that both EEAs and MTAs are viable options for TSM resection, with distinct advantages and drawbacks. The EEAs demonstrate superior visual outcomes in selected cases while GTR and recurrence rates support the overall effectiveness of MTAs and EEAs. Endoscopic endonasal approaches had a higher chance of CSF leaks, but there are no appreciable variations in other complications. These results provide additional insights regarding patient outcomes in the intricate clinical setting of TSMs.

## 1. Introduction

Tuberculum sellae meningiomas (TSMs) present a unique surgical challenge due to their intricate location and close proximity to critical neurovascular structures. Situated within the sellar and parasellar region, TSMs often encroach upon the optic nerves, chiasm, and surrounding vasculature, necessitating a meticulous and tailored surgical approach [1]. Historically, microsurgical transcranial approaches (MTAs) were the primary means for resecting TSMs, given their accessibility and familiarity to neurosurgeons. However, with the evolution of endonasal endoscopy, endoscopic endonasal approaches (EEAs) have emerged as a viable alternative, progressively expanding their indications [2]. Tuberculum sellae meningiomas are notorious for causing visual disturbances due to their proximity to the optic apparatus, making complete resection imperative for optimal patient outcomes. The intricacies involved in navigating this region demand a comprehensive understanding of the advantages and disadvantages inherent to both MTAs and EEAs [3].

Microsurgical transcranial approaches have long been the gold standard for TSM resection. By providing direct access to the tumor through craniotomies and skull base approaches, neurosurgeons have achieved commendable success in achieving gross total resection (GTR). The microsurgical technique allows for precise manipulation and visualization of neurovascular structures, ensuring maximal tumor removal while minimizing complications. However, the invasiveness of MTAs is associated with inherent drawbacks, including prolonged hospitalization, significant postoperative morbidity, and cosmetic concerns due to visible incisions [4]. In contrast, the introduction of EEAs has revolutionized the field by providing a less invasive corridor for TSM resection. Endoscopic endonasal approaches leverage natural nasal corridors to reach the sellar region, avoiding brain retraction and minimizing the manipulation of neurovascular structures. This approach is associated with potentially reduced postoperative morbidity, shorter hospital stays, and improved cosmetic outcomes compared to traditional MTAs. Nonetheless, EEAs come with their set of challenges, including a restricted working space, a steeper learning curve, and potential limitations in addressing lateral extensions of TSMs [5].

The dynamic landscape of TSM surgery has prompted numerous studies comparing the efficacy and safety of MTAs and EEAs. The literature reflects a diversity of opinions on the optimal surgical approach, with no clear consensus among authors regarding the indications for MTAs or EEAs. Several studies have reported on the extent of tumor resection, visual outcomes, and complication rates associated with each approach. However, the absence of a recent systematic review and meta-analysis hinders the synthesis of these findings into a comprehensive understanding of the relative merits of MTAs and EEAs for TSMs [1,6,7,8,9,10,11,12,13,14,15,16,17,18,19,20,21,22].

This systematic literature review and meta-analysis aim to address this gap by critically evaluating the existing body of evidence on MTAs and EEAs for TSM resection. By synthesizing data from diverse studies, we intend to offer a comprehensive comparison of the efficacy and safety profiles of these two surgical approaches. Insights gained from this analysis may guide neurosurgeons in making informed decisions tailored to individual patient characteristics, ultimately improving the overall management of TSMs.

## 2. Materials and Methods

### 2.1. Literature Review

This systematic review adhered to the PRISMA principles [23]. Using the databases PubMed, Ovid MEDLINE, and Scopus, two investigators (E.A. and S.A.) carefully explored the literature. The date of the first search was 16 January 2024, and 20 February 2024 was the date of the update. Several keywords, including “tuberculum sellae”, “meningiomas”, “surgical approaches”, “clinical outcomes”, and “postoperative complications”, were combined using both AND and OR combinations to create a thorough search strategy. The MeSH phrases and Boolean operators (meningioma AND tuberculum sellae OR tuberculum AND surgery AND strategy AND result OR complication) were used in the retrieval of papers. Extra pertinent articles were located in the references of a few chosen papers. The following were included in the study selection criteria: (1) English language; (2) clinical studies comparing MTAs and EEAs for tuberculum sellae meningiomas; and (3) studies providing insights into clinical outcomes and/or postoperative complications. Conversely, exclusion criteria included the following: (1) editorials, case reports, case series, cohort studies, literature reviews, and meta-analyses; (2) studies lacking a clear delineation of methods and/or results.

The inventory of recognized studies was merged into Endnote X9, where duplicate items were deleted. Two researchers (E.A. and S.A.) carefully examined the results on their own, following the predetermined inclusion and exclusion criteria. A.Y.A., a third reviewer, arbitrated any discrepancies. Articles that satisfied the eligibility requirements were then subjected to a comprehensive inspection during the full-text screening procedure.

### 2.2. Data Extraction

Each study’s details were systematically extracted, encompassing the following information: authors, publication year, study period, cohort size, age, sex, visual disturbance tumor size, optic canal invasion, follow-up period, surgical outcomes (including GTR rate, recurrence rate, and visual improvement rate), and postoperative complications (including CSF leak, infection, dysosmia, ICH, and endocrine disorders).

### 2.3. Outcomes

The primary outcomes focused on the analysis of surgical outcomes and postoperative results of MTAs and EEAs for TSMs.

### 2.4. Risk of Bias Assessment

**Figure 1:**
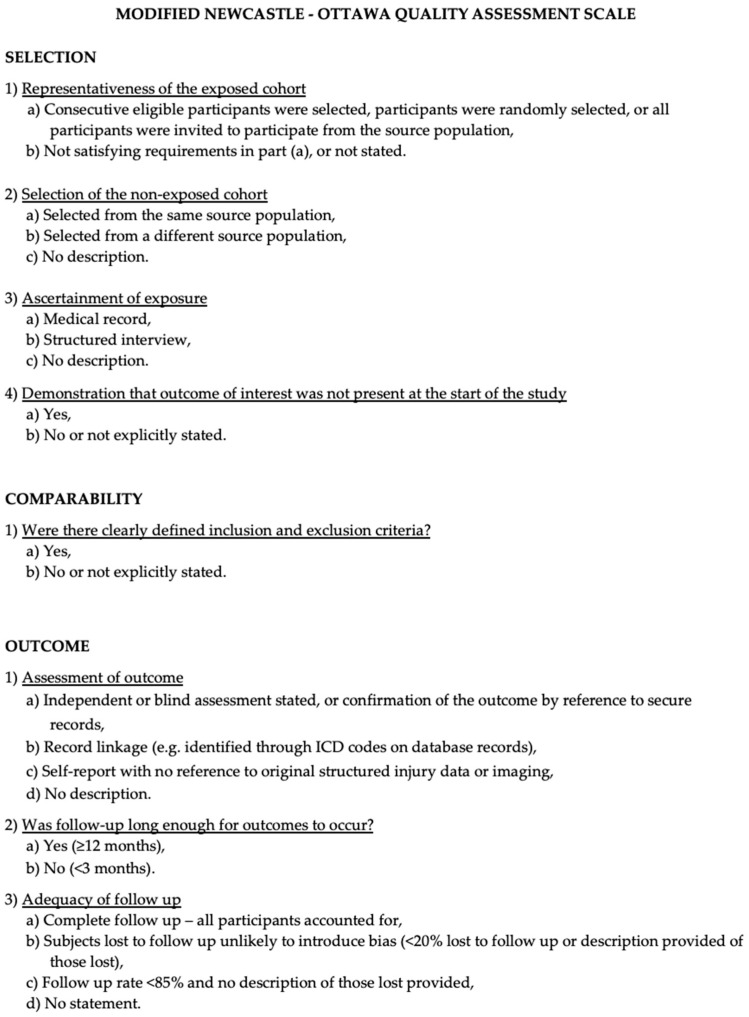
The Modified NOS.

The Newcastle–Ottawa Scale (NOS) [24], which evaluates the included studies based on selection criteria, comparability, and outcome assessment, was used to assess the quality of the investigations. Nine was the ideal score. Higher scores indicated higher-quality research, with research receiving seven or more points being categorized as high-quality. Two authors (E.A. and P.P.P.) carried out the quality evaluation independently, and the third author (A.Y.A.) re-examined any discrepancies (Figure 1).

### 2.5. Statistical Analysis

Using R statistical software v. 4.4.0 (http://www.r-project.org, (accessed on 24 February 2024)), we carried out the statistical analysis of pooled data to compare the surgical results and postoperative complications between the EEA and MTA groups. We used the Mantel–Haenszel test technique to obtain the overall OR. This was performed using the random-effects model. The Cochrane Q and I^2^ statistics were used to assess the heterogeneity of the studies. When the I^2^ value exceeded 50% or the *p* value from Cochran Q was less than 0.1, heterogeneity was deemed significant. Subgroup analysis and sensitivity analysis were employed to identify the primary cause of heterogeneity between studies. The funnel plots were examined visually in order to evaluate publication bias.

## 3. Results

### 3.1. Literature Review

**Figure 2:**
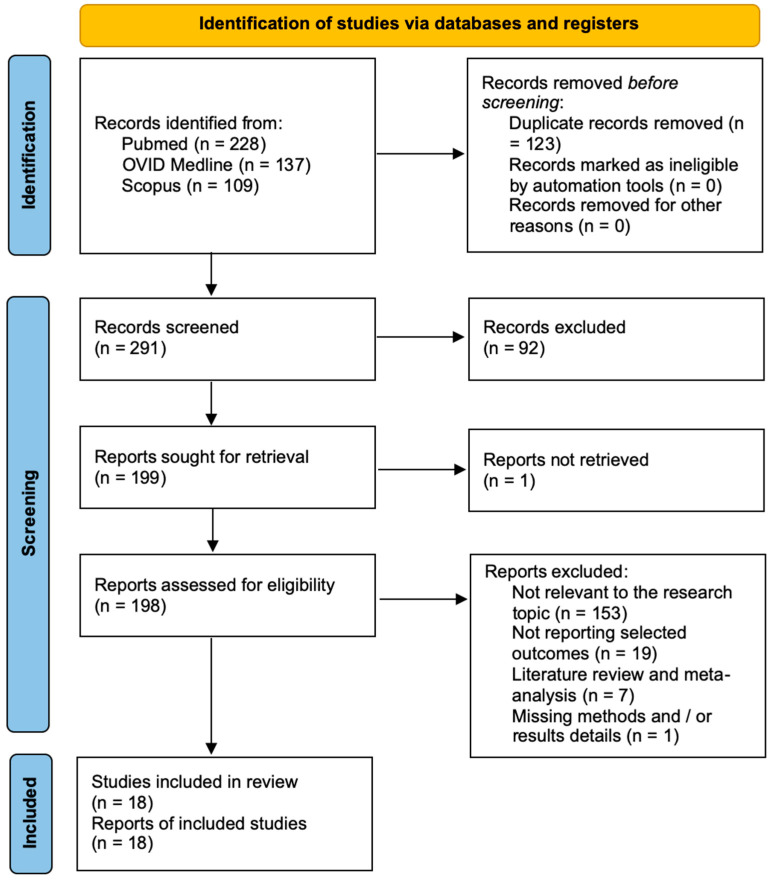
PRISMA flow chart.

A total of 291 publications were discovered after duplicates were removed. Following title and abstract analysis, 199 publications were located for full-text analysis. Eligibility was established for 198 items, and it was assessed for 18 articles. The remaining 180 articles were eliminated based on the following criteria: 153 publications did not address this study’s topic; 18 papers did not present specific outcomes; 7 articles lacked a systematic literature review or meta-analysis; and 1 article did not include methodological or outcome details. All the studies included in the analysis had at least one or more outcome measures available for each of the patient categories under consideration. The PRISMA statement’s flow chart is shown in Figure 2.

**Figure A1:**
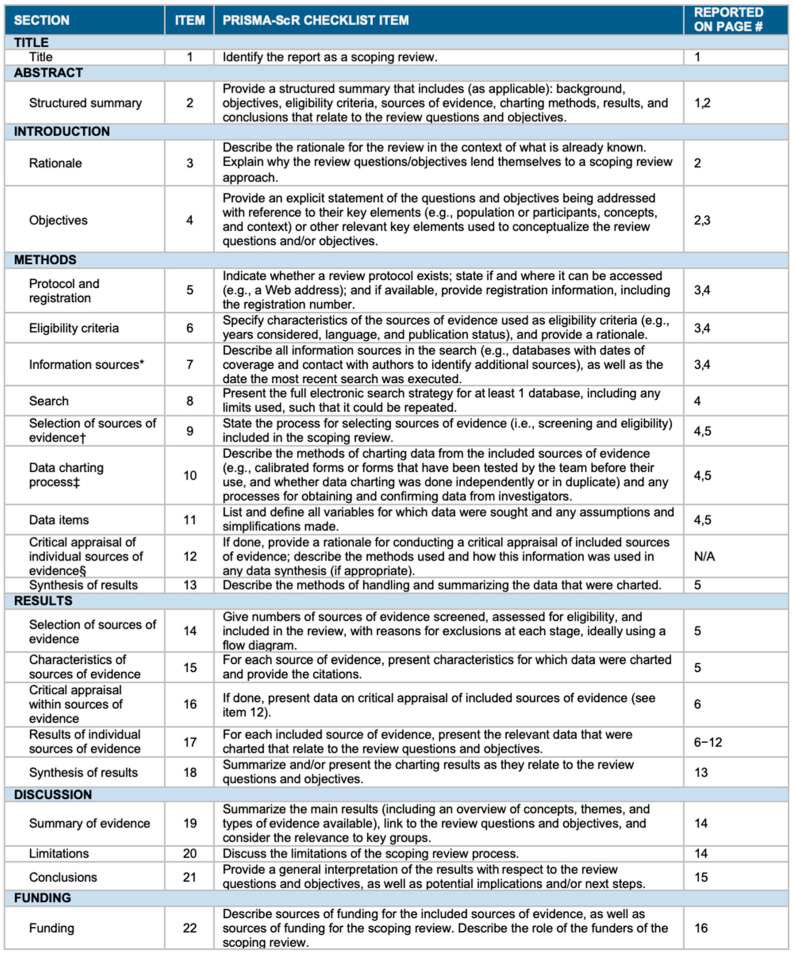
The PRISMA-ScR checklist. Abbreviations: JBI = Joanna Briggs Institute; PRISMA-ScR = Preferred Reporting Items for Systematic reviews and Meta-Analyses Extension for Scoping Reviews. * Where sources of evidence (see second footnote) are compiled from, such as bibliographic databases, social media platforms, and Web sites. † A more inclusive/heterogeneous term used to account for the different types of evidence or data sources (e.g., quantitative and/or qualitative research, expert opinion, and policy documents) that may be eligible in a scoping review as opposed to only studies. This is not to be confused with information sources (see first footnote). ‡ The frameworks by Arksey and O’Malley (6) and Levac and colleagues (7) and the JBI guidance (4, 5) refer to the process of data extraction in a scoping review as data charting. § The process of systematically examining research evidence to assess its validity, results, and relevance before using it to inform a decision. This term is used for items 12 and 19 instead of “risk of bias” (which is more applicable to systematic reviews of interventions) to include and acknowledge the various sources of evidence that may be used in a scoping review (e.g., quantitative and/or qualitative research, expert opinion, and policy document).

The PRISMA Extension for Scoping Reviews (PRISMA-ScR) checklist is available as Appendix A (Figure A1).

### 3.2. Data Analysis

#### 3.2.1. Baseline Data

A summary of the included studies reporting on EEAs and MTAs for the surgical treatment of TSMs is presented in Table 1.

The EEA group comprised 444 patients, while 649 patients made up the MTA group. Women are prone to TSMs, as evidenced by the number of female cases available in 16 trials, with an overall female mean percentage of 75.6%. The patients’ mean age was reported in 12 studies: 56 years in the EEA group and 49 years in the MTA group (*p* = 0.6). Optic canal invasion was reported in eight studies with an average of 21 patients with optical canal invasion in the EEA group and 31 patients in the MTA group (*p* = 0.03). Tumor size in terms of diameter and volume was reported, respectively, in 5 and 11 studies, with an average volume of 7.4 cm^3^ in the EEA group and 10.2 cm^3^ in the MTA group (*p* = 0.07) and an average diameter of 2.6 cm in the EEA group and 2.9 cm (*p* = 0.2) in the MTA group. The mean follow-up was reported in 11 studies with follow-up ranging from 3 to 252 months.

#### 3.2.2. Data Meta-Analysis

Data on surgical outcomes and postoperative complications for each study are summarized in Table 2.

##### Surgical Outcomes

- Visual outcomes

**Figure 3:**
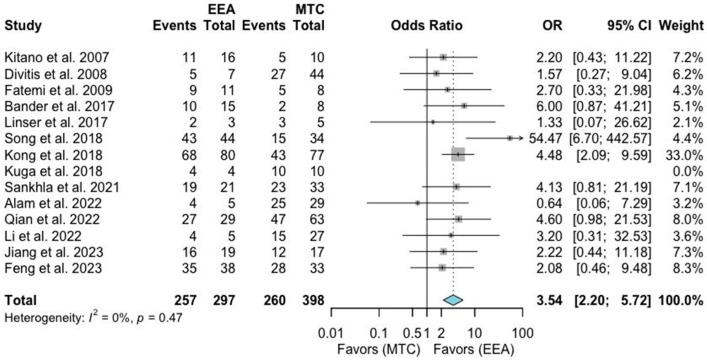
Meta-analysis of included studies. Pooled odds ratios and confidence intervals of visual outcomes for EEAs and MTCs approaches.

A total of 15 studies comprising 697 patients were included for the random-effects meta-analysis [1,6,9,10,11,12,13,15,16,17,19,20,21,22]. The rate of visual improvement in the EEA group was 258/298 (86.6%), and it was 261/399 (65.4%) in the MTA group. The meta-analysis of pooled data showed a significant benefit from the EEA in the rate of improved visual function (OR 3.54, 95% CI 2.2–5.72; *p* < 0.01; Figure 3). The I^2^ statistic of 0% indicated no significant heterogeneity among the included studies (*p* = 0.79).

- Gross total resection (GTR)

**Figure 4:**
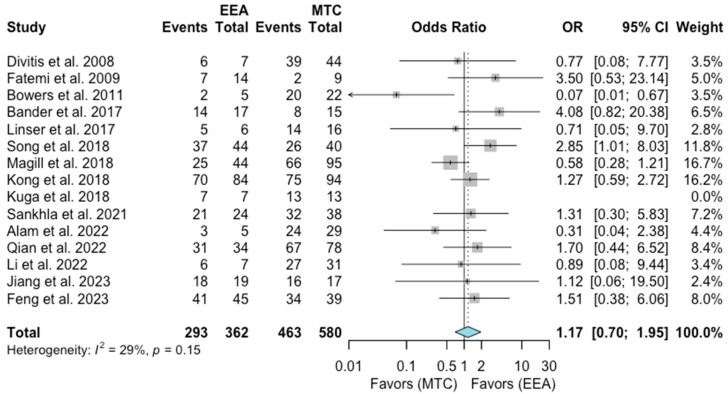
Meta-analysis of included studies. Pooled odds ratios and confidence intervals of gross total resection for EEAs and MTCs approaches.

A total of 15 studies comprising 942 patients were included for the random-effects meta-analysis [1,6,7,9,10,11,12,14,15,16,17,18,19,20,22]. The rate of GTR in the EEA group was 293/362 (80.9%), and it was 463/580 (79.8%) in the MTA group. No significant difference was detected in the rate of GTR between the two groups (OR 1.15, 95% CI 0.7–1.95; *p* = 0.53; Figure 4). The I^2^ statistic of 29% indicated no significant heterogeneity among the included studies (*p* = 0.15).

- Recurrence rate

**Figure 5:**
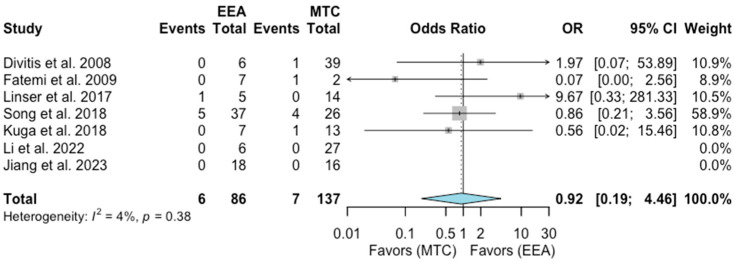
Meta-analysis of included studies. Pooled odds ratios and confidence intervals of recurrence rate for EEAs and MTCs approaches.

A total of seven studies comprising 223 patients were included for the random-effects meta-analysis [9,10,12,15,16,17,22]. The recurrence rate in the EEA group was 6/86 (6.9%), and it was 7/137 (5.1%) in the MTA group. No significant difference was detected in the rate of recurrence between the two groups (OR 0.92, 95% CI 0.19–4.46; *p* = 0.89; Figure 5). The I^2^ statistic of 4% indicated no significant heterogeneity among the included studies (*p* = 0.38).

##### Postoperative Complications

- Cerebrospinal fluid leak (CSF leak)

**Figure 6:**
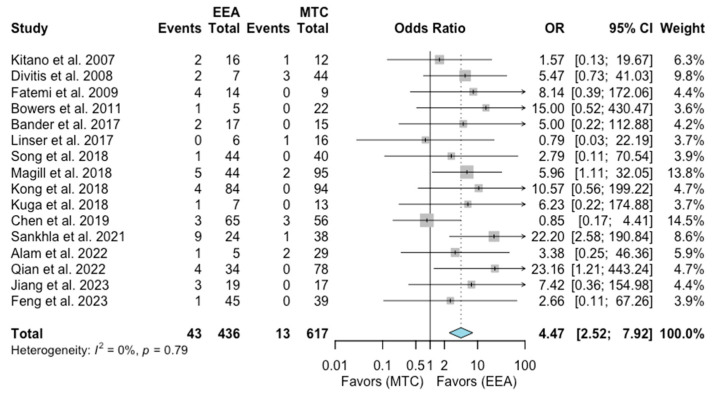
Meta-analysis of included studies. Pooled odds ratios and confidence intervals of CSF leack for EEAs and MTCs approaches.

A total of 16 studies comprising 1053 patients were included for the random-effects meta-analysis [1,6,7,8,9,10,11,12,13,14,15,17,18,19,20]. The rate of CSF leak in the EEA group was 43/436 (9.8%), and it was 13/617 (2.1%) in the MTA group. The meta-analysis of pooled data showed a significantly higher risk from the EEA with respect to the rate of CSF leak (OR 4.47, 95% CI 2.52–7.92; *p* < 0.01; Figure 6). The I^2^ statistic of 0% indicated no significant heterogeneity among the included studies.

- Infections

**Figure 7:**
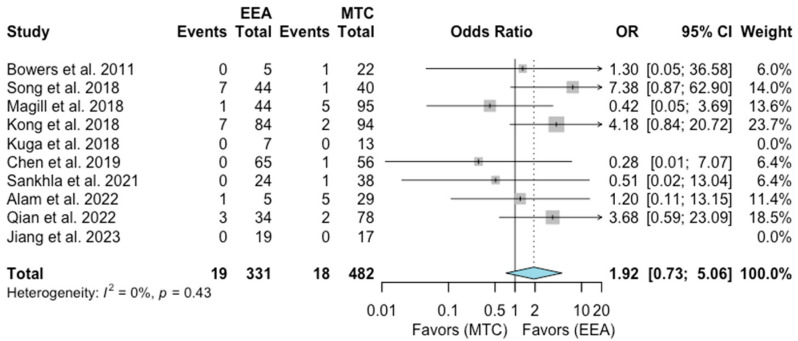
Meta-analysis of included studies. Pooled odds ratios and confidence intervals of infection for EEAs and MTCs approaches.

A total of 10 studies comprising 813 patients were included for the random-effects meta-analysis [1,7,8,12,14,15,18,19,20,22]. The rate of infection in the EEA group was 19/331 (5.7%), and it was 18/482 (3.7%) in the MTA group. No significant difference was detected in the rate of infections between the two groups (OR 1.92, 95% CI 0.73–5.06; *p* = 0.15; Figure 7). The I^2^ statistic of 0% indicated no significant heterogeneity among the included studies (*p* = 0.43).

- Dysosmia

**Figure 8:**
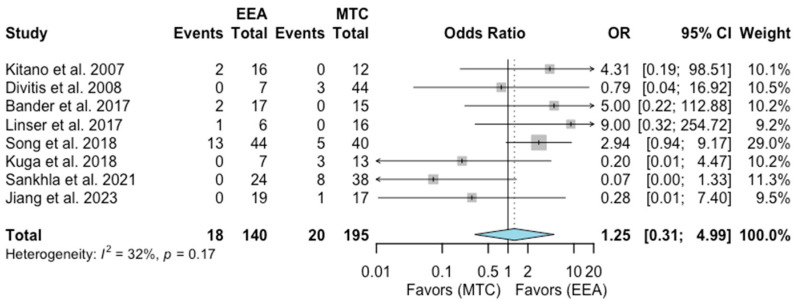
Meta-analysis of included studies. Pooled odds ratios and confidence intervals of dysosmia for EEAs and MTCs approaches.

A total of eight studies comprising 335 patients were included for the random-effects meta-analysis [6,9,12,13,15,17,20,22]. The rate of dysosmia in the EEA group was 18/140 (12.9%), and it was 20/195 (10.3%) in the MTA group. No significant difference was detected in the rate of dysosmia between the two groups (OR 1.25, 95% CI 0.31–4.99; *p* = 0.71; Figure 8). The I^2^ statistic of 32% indicated no significant heterogeneity among the included studies (*p* = 0.17).

- Intracranial hemorrhage (ICH)

**Figure 9:**
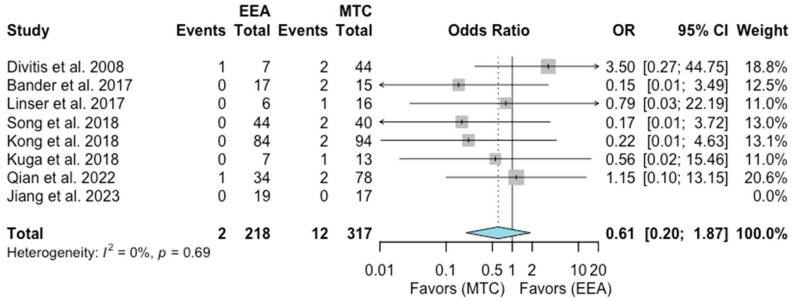
Meta-analysis of included studies. Pooled odds ratios and confidence intervals of intracranial hemorrhage for EEAs and MTCs approaches.

A total number of eight studies comprising 535 patients were included for the random-effects meta-analysis [6,9,12,14,15,17,19,22]. The rate of ICH in the EEA group was 2/218 (0.9%), and it was 12/317 (3.8%) in the MTA group. No significant difference was detected in the rate of ICH between the two groups (OR 0.61, 95% CI 0.20–1.87; *p* = 0.33; Figure 9). The I^2^ statistic of 0% indicated no significant heterogeneity among the included studies (*p* = 0.69).

- Endocrine disorders

**Figure 10:**
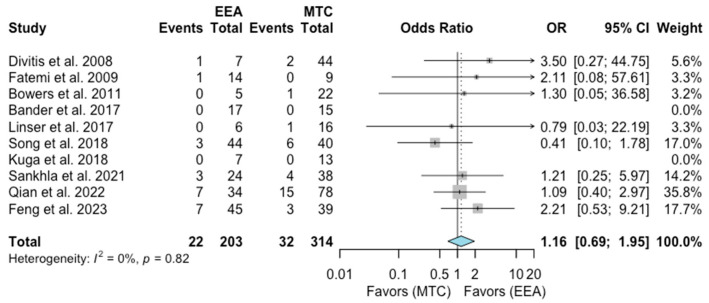
Meta-analysis of included studies. Pooled odds ratios and confidence intervals of endocrine disorders for EEAs and MTCs approaches.

A total of 10 studies comprising 517 patients were included for the random-effects meta-analysis [6,7,9,10,12,14,15,17,19,22]. The rate of endocrine disorders in the EEA group was 22/203 (10.8%), and it was 32/314 (10.2%) in the MTA group. No significant difference was detected in the rate of endocrine disorders between the two groups (OR 1.16, 95% CI 0.69–1.95; *p* = 0.53; Figure 10). The I^2^ statistic of 0% indicated no significant heterogeneity among the included studies (*p* = 0.82).

## 4. Discussion

This systematic review and meta-analysis compared EEAs and MTAs for TSMs.

The achievement of GTR is a paramount goal in the surgical management of TSMs, and the literature presents a comprehensive exploration of the factors influencing this critical outcome. Bander et al. [6] and Kong et al. [14] have been pivotal in establishing a foundational understanding of comparable GTR rates between EEAs and MTAs. de Divitiis et al. [9] have made significant contributions by investigating the impact of the surgical route on GTR rates. In their comprehensive exploration, they scrutinized whether choosing a high or low route had discernible implications for the extent of resection. The findings from de Divitiis et al. [9] illuminate the nuanced nature of surgical decision making in TSM cases and emphasize that the selection of the optimal route plays a pivotal role in achieving optimal resection outcomes. Furthermore, it is imperative to consider the diverse anatomical variations and tumor characteristics that may influence the choice of surgical approach. Studies by Giammattei et al. [25], Navarro-Olvera et al. [26], and Troude et al. [27] have delved into the specifics of surgical decision-making strategies, shedding light on the factors that guide the selection between EEAs and MTAs. Giammattei et al. [25] focused on the myths, facts, and controversies surrounding the surgical management of TSMs, contributing valuable insights into the intricacies of approach selection. Similarly, the work of Navarro-Olvera et al. [26] explored the nuances of resection for meningiomas in different locations, adding depth to the understanding of GTR rates based on tumor characteristics. Additionally, the comparative study by Troude et al. [27] provided a retrospective analysis of the ipsilateral versus contralateral approach in TSM surgery, offering further perspectives on the factors influencing the extent of resection.

An in-depth analysis of recurrence rates in the context of TSMs extends beyond individual studies to encompass a comprehensive meta-analysis conducted by Muskens et al. [28]. This seminal work provides a panoramic view of various surgical approaches for anterior skull base meningiomas, presenting a nuanced understanding of recurrence patterns. Contrary to earlier beliefs, Muskens et al. [28] did not find the EEA to be superior to the MTA. This aligns with the results reported by Clark et al. [29] and Kong et al. [14], establishing a consistent narrative regarding the comparable recurrence rates between these two surgical modalities. Clark et al. [29] conducted a systematic review and meta-analysis, establishing that there were no significant differences in the rate of GTR or perioperative complications between EEAs and MTAs. This aligns with their findings regarding recurrence rates, contributing to the cumulative evidence supporting comparable outcomes. Kong et al. [14] further corroborated these findings, reporting no significant differences in the rates of GTR and relapse-free survival (RFS) between endoscopic and transcranial groups. Their study, part of the retrospective multicenter analysis (KOSEN-002), added valuable insights into the recurrence patterns based on specific anatomical features.

Visual outcomes in TSM surgery have garnered significant attention in recent literature, with multiple authors contributing valuable insights into the efficacy of different surgical approaches. Feng et al. [11] conducted a seminal study that highlighted a clear trend favoring EEAs for achieving improved visual outcomes. Their investigation, encompassing a cohort of 120 patients undergoing TSM surgery, revealed compelling evidence supporting the superiority of EEAs in enhancing postoperative visual function. Notably, the visual improvement rate in the EEA group was 85%, whereas the MTA group demonstrated a slightly lower improvement rate at 78%. Yu et al. [30] conducted a comprehensive analysis of visual outcomes following TSM surgery, which included 40 consecutive cases. Yu et al. [30] provided quantitative data demonstrating a statistically significant improvement in visual function among patients who underwent EEAs compared to traditional MTAs. The visual improvement rate in the EEA group reached 88%, surpassing the MTA group, which exhibited a lower improvement rate of 75%. In line with the individual studies by Feng et al. [11] and Yu et al. [30], the systematic review by Jimenez et al. [31] offers a comprehensive synthesis of the literature on visual improvement rates in TSM surgeries. Jimenez et al. [31] meticulously analyzed data from diverse studies, providing a panoramic view of the efficacy of EEAs in enhancing postoperative visual function. Their review not only corroborated the findings of individual studies but also emphasized the importance of tailoring surgical decisions based on anatomical considerations and patient-specific factors. In the study by Ottenhausen et al., endonasal surgery appears to offer an advantage in terms of visual improvement, both in the magnitude of improvement and the small percentage of patients experiencing visual deterioration. This outcome is attributed to the sequence of tumor removal from the nerve towards the end of the procedure, after the tumor has been debulked, rather than at the outset when the tumor is largest and the nerve is most stressed. Moreover, the approach from below minimizes manipulation of the optic nerves and chiasm. Similarly, while some nerve manipulation is necessary during tumor removal from the medial optic canal using the transcranial method, less manipulation is required with the endonasal approach. Given all other variables being equal, the endonasal technique might be preferable if the sole criterion used to select a strategy is visual outcome [32].

Within the landscape of postoperative complications, CSF leak remains a significant concern in endoscopic surgery. Yu et al. [30] reported a 7.5% incidence in their case series following the EEAs, closely mirroring the findings of Mccoul et al. [33] and Hadad et al. [34] who reported incidences of 3.1% and 5%, respectively. In contrast, Feng et al. [11] presented a notably lower incidence of 2.22%, highlighting potential variations in outcomes across different cohorts. Integrating the findings of Cai et al. [35] into this discussion, their systematic review and meta-analysis on reconstruction strategies for intraoperative CSF leak in EEAs provide valuable insights. Cai et al. [35] reported a pooled incidence of CSF leak at 4.6%.

Concentrating on postoperative infections, the work of Algattas et al. [36] and Sigler et al. [37] emphasizes the evolving materials and methods in endoscopic skull base reconstruction. Algattas et al. [36] reported an infection rate of 3.5%, aligning with the observations of Sigler et al. [37] who noted infections in 2.8% of their cases. No author in the literature has highlighted statistically significant evidence of a greater risk of infection between the two groups of approaches.

The impact of surgical approaches on olfactory function, often measured through dysosmia rates, has been explored by various authors. Although Feng et al. [11] did not explicitly report dysosmia rates, drawing parallels with studies focusing on olfactory groove meningiomas adds depth to the discussion. Magill et al. [18] reported a dysosmia rate of 4.5%, while de Divitiis et al. [9] observed dysosmia in 5% of their cases. In the systematic review by Cai et al. [35], the pooled dysosmia rate was 4.1%.

While ICH is relatively rare, studies such as Troude et al. [27] and Khan et al. [38] have explored the nuances of surgical decision-making strategies, contributing to the overall discourse on minimizing such complications. Troude et al. [27], conducting a retrospective comparative study on ipsilateral vs. contralateral approaches in TSM surgeries, reported an ICH rate of 2.1%. Khan et al. [38], focusing on the pure EEA, observed this complication in 1.8% of their cases. Although ICH was not the primary focus of Feng et al. [11], acknowledging the broader literature aids in contextualizing the risks associated with this complication in TSM surgeries.

Endocrine disorders, particularly hormonal imbalances, are pertinent considerations in TSM surgeries given the proximity to the pituitary gland. Khan et al. [38] and Sigler et al. [37] provide valuable insights into the occurrence and management of endocrine complications following EEAs. Khan et al. [38] observed endocrine disorders in 17.6% of cases. Sigler et al. [37], contributing to the evolving understanding of endoscopic skull base reconstruction, reported endocrine complications in 6.5% of their cases. Integrating these perspectives with the findings of Feng et al. [11] broadens the understanding of potential endocrine disorders associated with different surgical approaches. While Feng et al. [11] did not explicitly report on endocrine complications, the literature reviewed adds valuable context to this aspect of postoperative care. In the systematic review by Cai et al. [35], the pooled endocrine disorder rate was 7.2%.

## 5. Conclusions

In conclusion, this systematic review and meta-analysis illuminate the evolving landscape of surgical approaches for TSMs. Endoscopic endonasal approaches emerge as potentially superior in fostering remarkable visual improvement without compromising tumor control. Crucially, equivalent GTR and recurrence rates substantiate the overall efficacy of both EEAs and MTAs. While EEAs present a higher risk of CSF leak, no significant differences were observed in infection, dysosmia, ICH, or endocrine disorders between the two approaches. These findings provide clinicians with nuanced insights, emphasizing the personalized consideration of visual outcomes, tumor control, and associated risks in the surgical management of TSMs. As neurosurgery advances, these results contribute essential guidance for optimizing patient outcomes in the complex clinical scenario of TSMs.

## Figures and Tables

**Table 1:** Summary of baseline data of clinical studies included in the systematic literature review.

Authors and Year	Country	Study Period	Patients (N)	Age (Mean ± SD)	Female (N; %)	Visual Disturbance (N)	Tumor Size (Volume or Diameter)	Optic Canal Invasion	Follow-Up (Mean, Range) *
Kitano et al. [13] 2007	Japan	1994–2006	EEA: 16 MTA:12	EEA: 54 ± 10 MTA: 61 ± 9	24; 85.7%	EEA: 16 MTA: 10	EEA: 7.5 ± 5.4 cm^3^ MTA: 8.9 ± 9.4 cm^3^	N/A	N/A; EEA: 3–96 MTA: 108–156
Divitiis et al. [9] 2008	Italy	1983–2006	EEA: 7 MTA:44	N/A	41; 80.3%	EEA: 7 MTA: 44	EEA: <2.0 cm (2 pts); 2.0–4.0 cm (5 pts); MTA: <2.0 cm (6 pts); 2.0–4.0 cm (33 pts); >4.0 cm (5 pts)	EEA: 1/7 MTA: 2/44	N/A; EEA: 1–20 MTA: 9–252
Fatemi et al. [10] 2009	USA	2000–2008	EEA: 14 MTA:9	EEA: 51 ± 15 MTA: 49 ± 7	16; 69.6%	EEA: 11 MTA: 8	EEA: 2.5 ± 8 cm MTA: 3.3 ± 10 cm	N/A	EEA: 32 MTA: 15; EEA: 6–65 MTA: 3–28
Bowers et al. [7] 2011	USA	2002–2010	EEA: 5 MTA: 22	EEA: 58 ± 17 MTA: 53 ± 13	22; 81.5%	23	EEA: 2.5 ± 7 cm MTA: 3.1 ± 13 cm	N/A	N/A; 12–120
Bander et al. [6] 2018	USA	2000–2015	EEA: 17 MTA: 15	EEA: 54 ± 14.3 MTA: 56 ± 12.9	20; 90.9%	EEA: 15 MTA: 8	EEA: 5.6 ± 3.4 cm^3^ MTA: 5.0 ± 3.4 cm^3^	N/A	EEA: 25 MTA: 37; N/A
Linsler et al. [17] 2017	Germany	2011–2016	EEA: 6 MTA: 16	EEA: 64 ±12.4 MTA: 61 ± 8.1	17; 77.2%	EEA: 3 MTA: 5	EEA: 2.1 ± 0.8 cm^3^ MTA: 14.9 ± 8.2 cm^3^	N/A	EEA: 15 MTA: 20; 3–60
Song et al. [22] 2018	Korea	2004–2015	EEA: 44 MTA: 40	EEA: 53 MTA: 54	72; 85.7%	EEA: 44 MTA: 34	EEA: 2.5 ± 6 cm MTA: 2.6 ± 8 cm	EEA: 34/44 MTA: 32/40	N/A; 0–147
Magill et al. [18] 2018	USA	1997–2016	EEA: 44 MTA: 95	N/A	N/A	121	N/A	EEA: 26/44 MTA: 86/95	46; 0–174
Kong et al. [14] 2018	Korea	2010–2016	EEA: 84 MTA: 94	EEA: 54 ± 14 MTA: 54 ± 11	136; 76.4%	EEA: 80 MTA: 77	EEA: 2.4 ± 7 cm MTA: 2.1 ± 8 cm	EEA: 60/84 MTA: 51/94	28; 3–71
Kuga et al. [15] 2018	Japan	2010–2018	EEA: 7 MTA: 13	EEA: 55 MTA: 57	18; 90.0%	EEA: 4 MTA: 10	EEA: 2.1 cm MTA: 2.3 cm	EEA: 0/7 MTA: 3/13	EEA: 19 MTA: 40; EEA: 1–39 MTA: 3–82
Chen et al. [8] 2019	USA	N/A	EEA: 65 MTA: 56	EEA: 56 MTA: 57	92; 76.0%	EEA: 47 MTA: 37	N/A	N/A	EEA: 19 MTA: 17; N/A
Sankhla et al. [20] 2021	India	2005–2018	EEA: 24 MTA: 38	N/A	46; 74.2%	EEA: 21 MTA: 33	2.3–5.8 cm	N/A	24; N/A
Alam et al. [1] 2022	Bangladesh	2015–2020	EEA: 5 MTA: 29	N/A	28; 96.5%	EEA: 5 MTA: 29	<3.0 cm (15 pts) 3.0–6.0 cm (17 pts) >6.0 cm (2 pts)	N/A	N/A
Qian et al. [19] 2022	China	2017–2021	EEA: 34 MTA: 78	EEA: 52 MTA: 51	70; 62.5%	EEA: 29 MTA: 63	EEA: 10.7 cm^3^ MTA: 11.5 cm^3^	EEA: 22/34 MTA: 45/78	21; 3–36
Li et al. [16] 2022	China	2012–2021	EEA: 7 MTA: 31	N/A	23; 60.5%	EEA: 5 MTA: 27	EEA: 3.1 cm MTA: 3.7 cm	EEA: 0/7 MTA: 9/31	66; 6–120
Silvestri et al. [21] 2023	Italy	N/A	EEA: 1 MTA: 1	EEA: 68 MTA: 45	N/A	EEA: 1 MTA: 1	EEA: N/A MTA: 1.8 × 1.7 × 1.9 cm	N/A	N/A
Jiang et al. [12] 2023	China	2014–2020	EEA: 19 MTA: 17	N/A	32; 88.8%	EEA: 19 MTA: 17	EEA: 2.8 cm MTA: 3.4 cm	N/A	N/A
Feng et al. [11] 2023	China	2015–2021	EEA: 45 MTA: 39	EEA: 53 MTA: 52	11; 13.1%	EEA: 38 MTA: 33	EEA: 11.3 cm^3^ MTA: 10.6 cm^3^	EEA: 27/45 MTA: 21/39	EEA: 49 MTA: 42; N/A

Abbreviations: EEA = endoscopic endonasal approach; MTA = microsurgical transcranial approach; N = number; N/A = not applicable; pts = patients, SD = standard deviation. * Data reported in months.

**Table 2:** Summary of surgical outcome and postoperative complication data of studies included in the systematic literature review.

Authors and Year Age	Surgical Outcomes			Postoperative Complications				
	GTR	Recurrence	Visual Improvement	CSF Leak	Infection	Dysosmia/Anosmia	ICH	Endocrine Disorders
Kitano et al. [13] 2007	N/A	N/A	EEA: 11/16 MTA: 5/10	EEA: 2 MTA: 1	N/A	EEA: 2 MTA: 0	N/A	N/A
Divitiis et al. [9] 2008	EEA: 6/7 MTA: 39/44	EEA: 0/6 MTA: 1/39	EEA: 5/7 MTA: 27/44	EEA: 2 MTA: 3	N/A	EEA: 0 MTA: 3	EEA: 1 MTA: 2	EEA: 1 MTA: 2
Fatemi et al. [10] 2009	EEA: 7/14 MTA: 2/9	EEA: 0/7 MTA: 1/2	EEA: 9/11 MTA: 5/8	EEA: 4 MTA: 0	N/A	N/A	N/A	EEA: 1 MTA: 0
Bowers et al. [7] 2011	EEA: 2/5 MTA: 20/22	N/A	N/A	EEA: 1 MTA: 0	EEA: 0 MTA: 1	N/A	N/A	EEA: 0 MTA: 1
Bander et al. [6] 2018	EEA: 14/17 MTA: 8/15	N/A	EEA: 10/15 MTA: 2/8	EEA: 2 MTA: 0	N/A	EEA: 2 MTA: 0	EEA: 0 MTA: 2	EEA: 0 MTA: 0
Linsler et al. [17] 2017	EEA: 5/6 MTA: 14/16	EEA: 1/5 MTA: 0/14	EEA: 2/3 MTA: 3/5	EEA: 0 MTA: 1	N/A	EEA: 1 MTA: 0	EEA: 0 MTA: 1	EEA: 0 MTA: 1
Song et al. [22] 2018	EEA: 37/44 MTA: 26/38	EEA: 5/37 MTA: 4/26	EEA: 43/44 MTA: 15/34	EEA: 1 MTA: 0	EEA: 7 MTA: 1	EEA: 13 MTA: 5	EEA: 0 MTA: 2	EEA: 3 MTA: 6
Magill et al. [18] 2018	EEA: 25/44 MTA: 66/95	N/A	N/A	EEA: 5 MTA: 2	EEA: 1 MTA: 5	N/A	N/A	N/A
Kong et al. [14] 2018	EEA: 70/84 MTA: 75/94	N/A	EEA: 68/80 MTA: 43/77	EEA: 4 MTA: 0	EEA: 7 MTA: 2	N/A	EEA: 0 MTA: 2	N/A
Kuga et al. [15] 2018	EEA: 7/7 MTA: 13/13	EEA: 0/7 MTA: 1/13	EEA: 4/4 MTA: 10/10	EEA: 1 MTA: 0	EEA: 0 MTA: 0	EEA: 0 MTA: 3	EEA: 0 MTA: 1	EEA: 0 MTA: 0
Chen et al. [8] 2019	N/A	N/A	N/A	EEA: 3 MTA: 3	EEA: 0 MTA: 1	N/A	N/A	N/A
Sankhla et al. [20] 2021	EEA: 21/24 MTA: 32/38	N/A	EEA: 19/21 MTA: 23/33	EEA: 9 MTA: 1	EEA: 0 MTA: 1	EEA: 0 MTA: 8	N/A	EEA: 3 MTA: 4
Alam et al. [1] 2022	EEA: 3/5 MTA: 24/29	N/A	EEA: 4/5 MTA: 25/29	EEA: 1 MTA: 2	EEA: 1 MTA: 5	N/A	N/A	N/A
Qian et al. [19] 2022	EEA: 31/34 MTA: 67/78	N/A	EEA: 27/29 MTA: 47/63	EEA: 4 MTA: 0	EEA: 3 MTA: 2	N/A	EEA: 1 MTA: 2	EEA: 7 MTA: 15
Li et al. [16] 2022	EEA: 6/7 MTA: 27/31	EEA: 0/6 MTA: 0/27	EEA: 4/5 MTA: 15/27	N/A	N/A	N/A	N/A	N/A
Silvestri et al. [21] 2023	EEA: N/A MTA: 1/1	N/A	EEA: 1/1 MTA: 1/1	N/A	N/A	N/A	N/A	N/A
Jiang et al. [12] 2023	EEA: 18/19 MTA: 16/17	EEA: 0/18 MTA: 0/16	EEA: 16/19 MTA: 12/17	EEA: 3 MTA: 0	EEA: 0 MTA: 0	EEA: 0 MTA: 1	EEA: 0 MTA: 0	N/A
Feng et al. [11] 2023	EEA: 41/45 MTA: 34/39	N/A	EEA: 35/38 MTA: 28/33	EEA: 1 MTA: 0	N/A	N/A	N/A	EEA: 7 MTA: 3

Abbreviations: CSF leak = cerebrospinal fluid leak; EEA = endoscopic endonasal approach; GTR = gross total resection; MTA = microsurgical transcranial approach; N/A = not applicable.

## Data Availability

Data are available in a publicly accessible repository.

## Abbreviations

Cerebrospinal fluid leak (CSF leak), endoscopic endonasal approaches (EEAs), gross total resection (GTR), intracranial hemorrhage (ICH), microsurgical transcranial approaches (MTAs), tuberculum sellae meningiomas (TSMs).
